# The roles of T cells in psoriasis

**DOI:** 10.3389/fimmu.2023.1081256

**Published:** 2023-10-24

**Authors:** Peng Zhang, Yuwen Su, Siying Li, Hui Chen, Ruifang Wu, Haijing Wu

**Affiliations:** Department of Dermatology, Hunan Key Laboratory of Medical Epigenomics, The Second Xiangya Hospital of Central South University, Changsha, Hunan, China

**Keywords:** psoriasis, T lymphocytes, cytokines, targeted therapies, immunology

## Abstract

Psoriasis is a recurring inflammatory skin condition characterized by scaly, red patches on the skin. It affects approximately 3% of the US population and is associated with histological changes such as epidermal hyperplasia, increased blood vessel proliferation, and infiltration of leukocytes into the skin’s dermis. T cells, which are classified into various subtypes, have been found to play significant roles in immune-mediated diseases, particularly psoriasis. This paper provides a review of the different T lymphocyte subtypes and their functions in psoriasis, as well as an overview of targeted therapies for treating psoriasis.

## 1 Introduction

Psoriasis is a long-lasting and recurring inflammatory skin condition that affects nearly 3% of the US population (1). Psoriasis is characterized by raised, red patches on the skin covered by silver scales, and the histological features include an increase in the thickness of the skin’s top layer, proliferation of blood vessels in the skin’s dermis, and the presence of inflammatory leukocytes in the dermis (2). Psoriasis is not only a skin condition but is also associated with several comorbid conditions, including psoriatic arthritis (PsA), metabolic syndrome, cardiovascular diseases, and mental illnesses (3). Psoriasis is a dermatosis with multiple contributing factors. Genetics is one of the most significant factors, as almost 40% of individuals with psoriasis have a family history of the condition (4). Environmental factors may cause or aggravate psoriasis, such as stress, trauma, certain medications, infections, smoking and alcohol consumption (5). Originally, the pathogenesis of psoriasis was considered to be related with aberrant epidermal keratinocyte proliferation (6). There is a growing consensus that activated T lymphocytes play a crucial role in the development of psoriasis, which is supported by the successful treatment by cyclosporine. Cyclosporine has proved to be highly effective in the treatment of psoriasis, but its side effects (including renal toxic effects, hypertension, and an increased risk of malignant neoplasm) limit the use of cyclosporine as an acceptable long-term monotherapy for psoriasis (7). As a result, T cells are now considered to be the primary contributors to the pathology of psoriasis (8). And more precise and safe therapies are needed for patients with psoriasis.

Traditionally, T lymphocytes can be broadly classified into two groups: conventional T cells and innate-like T cells. Conventional T cells, such as CD4^+^ T helper lymphocytes and CD8^+^ cytotoxic T lymphocytes, recognize peptide antigens presented by major histocompatibility complex (MHC) molecules. In contrast, innate-like T cells, which include natural killer T cells, gamma delta T cells, and mucosal-associated invariant T cells, are involved in rapid immune responses that are not dependent on MHC expression (9). Here, we will review recent advances and roles of T lymphocytes in psoriasis as well as summarize currently targeted anti-psoriatic therapies.

## 2 Conventional adaptive T lymphocytes in psoriasis

### 2.1 CD4^+^ helper T cells in psoriasis

CD4^+^ helper T cells are so named because they express the CD4 glycoprotein on their surface. These cells become activated when presented with peptide antigens by MHC class II molecules, which are expressed on the surface of antigen-presenting cells and then produce cytokines that regulate or assist the immune system. Research has shown that CD4^+^ helper T cells are present in the dermal skin of individuals with psoriasis (10). The effects of CD4^+^ helper T cells in psoriasis can be observed in grafted skin with injected cells from psoriasis patients into graft sites on severe combined immunodeficiency disease (SCID) mice. These alterations in the skin demonstrate the functions of the CD4^+^ helper T cells in psoriasis (11).

These CD4^+^ helper T lymphocytes can be classified into Th1, Th2, Th17 and Th22 cells, performing different functions. Th1 cells can increase macrophages and cytotoxic T cells - mediated immune response by releasing interferon-γ (IFN-γ) and TNF-α, which are critical players in the development of psoriasis (12, 13) (**Table 1**). Studies found that cytokines secreted by T cells associated with psoriasis are almost Th1 related cytokines (IL-2, IFN-γ, and TNF-a), but not Th2 type cytokines (IL-4 and IL-10) (25). The data suggest that psoriasis is primarily influenced by Th1 cells. However, the use of humanized monoclonal antibodies that target IFN-γ for psoriasis therapy was not as successful as expected. This suggests that IFN-γ may have a more complex role in psoriasis than previously thought (26).

**Table 1:** Different subtypes of T cells and related cytokines in psoriasis.

T cell Type	Subtypes	Major cytokines released	Ref.
CD4^+^ helper T cells	Th1	IFN-γ, TNF-α, IL-2	9, 10
	Th2	IL-4, IL-10	11
	Th17	IL-17, TNF-α, IL-6, IL-21, IL-22	14, 15
	Th22	IL12, IL-13	16
CD8^+^ cytotoxic T cells	Tc1	IFN-γ, IL-2, TNF-α	17
	Tc17	TNF-α, IFN-γ, IL-17, IL-21, IL-22	18
	Tc22	IL-22	19
TRM cells	CD8^+^CD103^+^ TRM	IFN-γ, IL-17A, IL-22	20
Treg cells	/	IFN-γ, TNF-α, IL-17A	21
Innate-like T cells	NKT cells	/	/
	MAIT	IL-17	22
	γδ T cells	IL-17	23, 24

MAIT, Mucosal associated invariant T cells; NKT cells, Natural killer T cells; TRM cells, Tissue resident memory T cells; Treg cells, Regulatory T cells; γδ T cells, Gamma delta T cells.

T helper 17 cells (Th17 cells) are defined as a group of pro-inflammatory T helper cells secreting IL-17, but not IFN-γ (27). With the stimulus of IL-1 and IL-6, Th17 cells develop from naive CD4^+^ T cells and maintain on IL-23 produced by keratinocytes, Langerhans cells, Dendritic cells (DCs), and macrophages (14). IL-23 is a cytokine composed of two chains (p19 and p40) that are also shared by IL-12. An increasing amount of evidence suggests that the IL-23/Th17 axis and related cytokines play a significant role in psoriasis. In psoriatic skin, the expression levels of IL-23 are higher than in normal skin. Injecting IL-23 intradermally into murine models results in abnormal epidermal hyperplasia and keratinocyte proliferation (15). Biological agents that target IL-12/-23 and IL-23 have been effective in treating psoriasis patients (28, 29). On the other side, besides secreting IL-17, Th17 cells can also release TNF-α, IL-6, IL-21, and IL-22, participating in the development of psoriasis (30, 31). Clinical randomized trials targeting IL-17A and IL-17F antibodies in psoriasis have proven that these two cytokines can be used for therapeutic targets (16, 32, 33). A newly discovered subtype of T cells named Th22 cells produce IL-22/-13, but not IFN-γ, IL-4 or IL-17 (34). Similar with Th1/17 lymphocytes, the numbers of Th22 cells were upregulated in patients with psoriasis as well (35).

Recently, a newly discovered subtype of CD4^+^ T lymphocytes was defined as T follicular helper (Tfh) cells, exhibiting different functions compared to other T cell subsets. Tfh cells can express CXCR5, ICOS, PD-1, Bcl-6 and generate IL-21 as well (17). CXCR5 is the surface marker of Tfh cells and critical for Tfh-cell function (36). ICOS is required for Tfh-cell differentiation and PD-1 plays a role in regulating Tfh-cell function (37, 38). Tfh cells can be grouped as two subtypes — conventional and circulating Tfh cells. Conventional Tfh cells mainly contribute to the formation of germinal center, B cells differentiation and antibody production (18). Circulating Tfh cells are grouped as three subtypes by CXCR3 and CCR6, including Tfh 17 (CXCR3^-^CCR6^+^), Tfh1 (CXCR3^+^CCR6^-^), and Tfh2 (CXCR3^-^CCR6^-^) cells. Tfh 17 cells produce the Th17 cytokines (IL-17A and IL-22), while Tfh1 cells produce the Th1 cytokine (IFN-γ) and Tfh2 cells secrete IL-4, IL-5 and IL-13, which are Th2 cytokines (19). Changed in the balance of circulating Tfh cells are found to be related with autoimmune diseases such as systemic lupus erythematosus (SLE) (39), Henoch-Schonlein purpura (HSP) (40), rheumatoid arthritis (RA) (41), ankylosing spondylitis (AS) (42). Tfh cells can also participate in regulating B cell’s activation and function (43). In psoriasis, the components of circulating Tfh cells were also proven to be abnormal and Tfh 17 cell subset was increased and correlated with psoriasis area and severity index (PASI) score (20, 44). In addition, the frequency of Tfh17 cells diminished during the follow-up periods (20).

Generalized pustular psoriasis (GPP) is a subtype of psoriasis that is characterized by the infiltration of neutrophils into the epidermis, resulting in severe symptoms. Haskamp S et al. used single-cell RNA sequencing to analyze the transcriptomes of MPO-deficient patients in a stable disease state. The cell types were identified through multimodal reference mapping of the single-cell RNA sequencing data. The results indicated that the proportion of CD4^+^ cytotoxic T lymphocytes and other CD4^+^ effector cells were increased in GPP, while the frequency of naïve CD4^+^ T cells was significantly lower (45).

### 2.2 CD8^+^ cytotoxic T cells in psoriasis

For a long time, psoriasis was regarded as Th1 cell-mediated skin disorder (25). Gradually, growing evidence suggests that the IL-23/Th17 axis and IL-22/Th22 pathway play critical roles in psoriasis (46). In psoriatic skin lesions, CD4^+^ T cells are concentrated at upper dermis, while CD8^+^ T cells are mainly found in the epidermis (47). Expect CD4^+^ T lymphocytes, cytotoxic CD8^+^ T cells are also able to secret IL-2, IFN-γ, TNF-α, IL-17, and the IL-22 cytokine family, which are consecutively named as Tc1, Tc17, and Tc22 cells (48).

In psoriasis, Tc1 cells release IFN-γ, IL-2, and TNF-α, playing different roles during the development of psoriasis (49). Early in the psoriatic cascade, IFN-γ is capable of activating antigen-presenting cells (APCs) and keratinocytes to generate IL-22 and IL-1β, therefore enhancing cytokine storms in psoriasis (50). TNF-α in psoriasis is able to regulate APCs (51) and stimulate DCs to secret cytokines such as IL-23 (52). Besides, TNF-a can form strong synergies with other cytokines such as IL-17A (50) to amplify the inflammatory cascade and promote proliferation and chemotaxis of T cells to the lesional sites (51).

Through single-cell transcriptomics, researchers discovered two pathogenic cytotoxic type 17 T-cell (Tc17) subsets of CD8^+^ T cells in the psoriatic skin of 11 psoriasis patients and five healthy control individuals (53). In contrast to Th17 cells, Tc17 cells in psoriatic tissue can release TNF-α, IFN-γ (Th1-related cytokines) and IL-17/-21/-22 (Th17-related cytokines) (54). Moreover, Tc17 cells express CCR6 (the ligand for CCL20), which is necessary for epidermal homing of all CD8^+^ T cells (54). Except for the secretion of cytokine, CD8^+^IL-17^+^ T cells in psoriatic lesions can produce cytotoxic molecules (namely granzyme B) and decrease target cells in a T-cell receptor (TCR)/CD3-dependent way (54). However, the exact mechanism of cytotoxic target cell killing is still unclear.

Tc22 cells are another recently determined CD8^+^ T cell subtype in psoriasis and are predominately enriched in the psoriatic epidermis (55). Without IL-17 and IFN-γ, Th22 only secrete IL-22 in psoriatic skin (55). And those cells derived from Th17 and Tc17 cells are incapable of expressing IL-17A, and then develop into T-lymphocytes only producing IL-22 (55).

Approximately 30% of individuals with psoriasis may develop PsA, which is characterized by peripheral arthritis, enthesitis, and dactylitis (56). Studies have shown that the expansion of memory CD8^+^ T cells in the joints of individuals with PsA is significantly higher than in their peripheral blood. Additionally, CD8^+^ T cells have been observed in the synovial fluid of PsA patients in previous studies (57). The use of single-cell sequencing revealed that there is a greater presence of CD8^+^T cells in the synovial fluid of PsA patients that express CXCR3, a receptor that aids in tissue homing. Additionally, the ligands of CXCR3 (CXCL9 and CXCL10) were found to be expressed at higher levels, providing a molecular understanding of the cellular immune mechanism involved in PsA (58).

### 2.3 Tissue resident memory T cells (T_RM_ cells) in psoriasis

T_RM_ cells are defined as long-time survival, memory T cells and are abundant at epithelial and mucosal tissues (skin, mucosa, lung, brain, and gastrointestinal tract), which are different from recirculating central and effector memory T cells in transcriptional and functional aspects (59). CD49, CD69 and CD103 are regarded as the surface hall markers of T_RM_ cells, among which CD69 and CD103 belong to tissue-retention markers (60). Although anti-psoriasis biologic agents inhibiting TNF-a, IL-23/IL-12, IL-17A, and IL-17 receptors have achieved great therapeutic effectiveness compared to traditional treatments, the therapeutic effects are varied between patients and the skin lesions often recur once the biologics will be withdrawn (61). Interestingly, psoriasis plaques are prone to recur at the same anatomical locations (62).

Skin T_RM_ cells are thought to play roles in the pathogenetic process in psoriasis, which acts as a strong factor for lesion recurrence (63). CD8^+^CD103^+^ T_RM_ cells release psoriasis-associated cytokines such as IFN-γ, IL-17A, and IL-22, while CD4^+^CD103^+^ T_RM_ cells and CD8^+^CD103^-^ T_RM_ cells don’t secrete these cytokines (64). In addition, CD8^+^CD103^+^ T_RM_ cells can be further classified as two subtypes: CD49a^-^IL-17A^+^ and CD49a^+^IFNγ^+^, assumedly related to psoriasis and vitiligo, respectively (21). Furthermore, skin T_RM_ cells are also related to the clinical changes. For example, CD103^+^T_RM_ cells can release IL-17A in epidermis of subsided psoriasis and are related to early recurrence (65).

### 2.4 Regulatory T cells (Treg cells) in psoriasis

Treg cells are T cells that express biomarkers CD4, CD25 and FOXP3, and originated from the same cell lineage as naïve CD4^+^ T cells (66). Treg cells are immune-suppressive and suppress the induction and proliferation of effector T cells, playing roles in maintaining self-antigens tolerance (67).

Currently, the linkage between Treg frequency and psoriasis severity are ambiguous. Some studies revealed a downregulated trends of Tregs in the peripheral blood cells of psoriatic patients but their correlations with the severity of disease are not in accordance (68–70). However, there wasn’t significantly difference of circulating Treg frequency in other studies (22, 71–73). Compared to healthy skin, many studies have shown that there is an increased frequency of the infiltration of Treg cells into lesional skin (23, 72, 74–76). Furthermore, a higher frequency of Treg cells in dermis compared to epidermis in plaque psoriasis was observed (75) and the opposite results were also reported (23). Compared to non-lesional skin, Foxp3^+^ Tregs were downregulated in lesional skin of psoriatic patients with acute exacerbation but upregulated in chronic phase (73). Yan et al. reported that compared to normal skin, Foxp3^+^Tregs were increased in lesional skins of plaque psoriasis but decreased in guttate psoriasis (77).

It is indicated that Treg cells are dysfunctional in most patients with psoriasis. For example, CD4^+^ CD25^high^ Foxp3^+^ Treg cells from skin lesions and peripheral blood are unable to inhibit the proliferation of effector T cells (22). Tregs from peripheral blood of psoriatic patients are phosphorylated and responsible for abnormal activation of STAT3 pathway and over-expression of the pro-inflammatory cytokines (e.g. IFN-γ, TNF-α and IL-17A). The phosphorylation of STAT3 and subsequent dysfunction of Tregs are caused by IL-6, IL-21 and IL-23, suggesting the role of pro-inflammatory cytokine milieu in the impaired Treg dysfunction (78).

Besides, abnormal adenosine signaling pathway may weaken the suppressive function of Tregs in psoriasis. Normally, Treg cells express both CD39 and CD73 and use adenosine signaling for immune suppression (24, 79). However, the expression of CD73 expression of Treg cells from psoriatic patients is greatly decreased, and the CD73/AMPK pathway is inactive, therefore impairing the immunosuppressive function of Treg cells (80).

## 3 Innate-like T cells in psoriasis

### 3.1 Natural killer T cells (NKT cells)

NKT cells involve the receptor of NK and a TCR with α and β chains. According to the TCR type, NKT cells are sorted into type I and type II (81). The role of NKT cells in psoriasis development is not clearly understood. NKT cells are increased in skin lesions from psoriatic patients (82) and decreased after anti-psoriatic treatment (83). Although the current literature regarding the circulating NKT cells in psoriasis are inconsistent, transplantation of immune cells from patients with psoriasis into SCID mice with human skin xenografts develops psoriasis-like lesions and some of the infiltrating cells express NK receptors (84).

### 3.2 Mucosal associated invariant T cells (MAIT) in psoriasis

As a subtype of innate-like T cells, MAIT cells have a semi-invariant TCR including Vα7.2 and Jα33, Jα12 or Jα20 and predominately a Vβ13 (TRBV6) and Vβ2 (TRBV20) TCRβ chain (85). MAIT cells are mainly found in the skin of normal people and psoriatic patients (86). Data has shown that MAIT cells are the majority of the IL-17-producing CD8^+^ T cells in the blood after phorbol myristate acetate + ionomycin stimulation (87). The IL-17^+^ CD8^+^ T cells were accumulated in lesional skin of psoriasis and able to release TNFα, IL-17 and IFN-γ, resulting in promoting inflammatory process (54).

### 3.3 Gamma delta T cells (γδ T cells) in psoriasis

γδ T cells have a γδ TCR on their surface, which was first discovered by using the TCRγ gene sequence (88). Most T cells belong to αβ T cells which contain two glycoprotein chains, named α and β TCR chains. However, γδ T cells are made up of two chains, γ and δ chain, which are mainly distributed in skin, digestive, respiratory, and reproductive tracts (89).

In the skin, nearly 99% of the T cells belong to αβ T cells, while γδ T cells only make up 1% (63). γδ T cells were enriched in both epidermal and dermal skin in both human and mice (90). Humans γδ T cells are grouped by δ chain expression including the Vδ1, Vδ2, and Vδ3 subtypes (91), while murine γδ T cells can be sorted according to γ chain expression named Vγ1–Vγ7 subtypes (92). These γδ T cells in psoriatic skin are also named as skin homing Vγ9 Vδ2 T cells and their distribution varies in peripheral blood and skin tissue (93). Different from epidermal γδ T cells and conventional αβ T cells, derma γδ T cells are known as IL-17-producing γδ T cell and release IL-17 upon IL-23 stimulation in the skin (90, 94). Abnormal T cell activation, especially IFN-γ-producing Th 1 cells, has been known to play a vital part in psoriasis. Besides, IL-23/Th17 axis was confirmed to be impaired in the psoriasis development. In addition, the number of γδT cells in psoriasis is negatively related with the PASI score and neutrophil-lymphocyte ratio which is associated with the risk of cardiovascular event, implying the potential role of γδT cells in the development of psoriasis-related cardiovascular event (93). A Recent study showed that glutamine metabolism is a key factor for the generation of γδ T cells, which could be a possible target for γδ T cell-related disorders, such as psoriasis (95).

## 4 Targeted anti-psoriasis therapies

Targeted anti-psoriasis therapies namely biological agents have been developed with the recognition of TNF-a, IL-23/Th17 axis and IL-22/Th22 pathway, representing better efficacy as well as relative safety. Besides, there are several newly developed types of small molecule inhibitors (**Table 2**).

**Table 2:** Targeted anti-psoriasis therapies approved by FDA.

Targeted therapies	Types	Indications	Recommended dosages approved by FDA
TNF-α inhibitors			
Etanercept	a recombinant human TNF-α receptor fusion protein	Psoriatic arthritis	50 mg weekly
		Plaque psoriasis	50 mg twice weekly for 3 months, then 50 mg weekly
		Pediatric psoriasis	0.8 mg/kg weekly, with a maximum of 50 mg per week
Infliximab	a chimeric IgG1 monoclonal antibody	Psoriatic arthritis	5 mg/kg at 0, 2 and 6 weeks, then every 8 weeks
		Plaque psoriasis	
Adalimumab	a fully human monoclonal antibody of the IgG1 isotype	Psoriatic arthritis	40 mg every other week
		Plaque psoriasis	80 mg initial dose, followed by 40 mg every other week starting one week after the initial dose
Certolizumab pegol	a humanized, Fc free pegylated inhibitor of TNF-α	Psoriatic arthritis	400 mg at weeks 0, 2 and 4, then 200 mg every other week; 400 mg every 4 weeks for maintenance dosing can be considered
		Plaque psoriasis	400 mg every other week; for some patients with body weight 90 kg, 400 mg at weeks 0, 2, and 4, then 200 mg every other week may be considered
Golimumab	a humanized anti-TNF monoclonal antibody	Psoriatic arthritis	SC dosing: 50 mg every month IV dosing: 2 mg/kg at weeks 0 and 4, then every 8 weeks
IL-12/23 Inhibitors			
Ustekinumab	a humanized anti-p40 subunit monoclonal antibody	Plaque psoriasis	For patients 100 kg: 45 mg at weeks 0 and 4, then 45 mg every 12 weeks For patients >100 kg: 90 mg at weeks 0 and 4, then 90 mg every 12 weeks
		Psoriatic arthritis	45 mg at weeks 0 and 4, then 45 mg every 12 weeks
		Pediatric psoriasis	For patients <60 kg: 0.75 mg/kg at weeks 0 and 4, then 0.75 mg/kg every 12 weeks For patients 60–100 kg: 45 mg at weeks 0 and 4, then 45 mg every 12 weeks For patients >100 kg: 90 mg at weeks 0 and 4, then 90 mg every 12 weeks
Guselkumab	a humanized monoclonal antibody to IL-23	Plaque psoriasis	100 mg at Week 0, Week 4, and every 8 weeks thereafter
		Psoriatic arthritis	100 mg at Week 0, Week 4, and every 8 weeks thereafter
Tildrakizumab	a humanized monoclonal antibody to IL-23	Plaque psoriasis	100 mg at Weeks 0, 4, and every twelve weeks thereafter
Risankizumab	a humanized monoclonal antibody to IL-23	Plaque psoriasis	150 mg at Week0, Week 4, and every 12 weeks thereafter
		Psoriatic arthritis	150 mg at Week0, Week 4, and every 12 weeks thereafter
IL-17 inhibitors			
Secukinumab	a humanized monoclonal IL-17 antibody	Plaque psoriasis	300 mg at Weeks 0, 1, 2, 3, and 4 followed by 300 mg every 4 weeks
		Psoriatic arthritis	Coexistent moderate to severe plaque psoriasis: 300 mg by at Weeks 0, 1, 2, 3, and 4 followed by 300 mg every 4 weeks For other PsA patients: With a loading dosage is 150 mg at Weeks 0, 1, 2, 3, and 4 and every 4 weeks thereafter Without a loading dosage is 150 mg every 4 weeks
		Pediatric plaque psoriasis	at weeks 0, 1, 2, 3, and 4 followed by dosing every 4 weeks For patients < 50kg: 75mg every injection For patients ≥ 50 kg: 150mg every injection
		Pediatric psoriatic arthritis	at weeks 0, 1, 2, 3, and 4 and every 4 weeks thereafter. For patients weighing ≥ 15 kg and < 50 kg the recommended dose is 75 mg For patients weighing ≥ 50 kg the recommended dose is 150 mg
Ixekizumab	a humanized monoclonal IL-17 antibody	Plaque psoriasis	160 mg (two 80 mg injections) at Week 0, followed by 80 mg at Weeks 2, 4, 6, 8, 10, and 12, then 80 mg every 4 weeks
		Psoriatic arthritis	160 mg by (two 80 mg injections) at Week 0, followed by 80 mg every 4 weeks
		Pediatric plaque psoriasis	For patients >50 kg: 160mg (two 80 mg injections) at week 0, then 80 mg every 4 weeks For patients 25-50kg: 80mg at week 0, then 40 mg every 4 weeks For patients < 25kg: 40mg at week 0, then 20mg every 4 weeks
Brodalumab	a humanized anti-IL-17RA monoclonal antibody	Plaque psoriasis	210 mg at Weeks 0, 1, and 2 followed by 210 mg every 2 weeks
Tofacitinib	a JAK inhibitor	Psoriatic Arthritis	5 mg twice daily
Upadacitinib	a JAK inhibitor	Psoriatic Arthritis	15 mg once daily
Deucravacitinib	a TYK2 inhibitor	Plaque psoriasis	6 mg once daily
Apremilast	a PDE4 inhibitor	Plaque psoriasis	Day 1: 10 mg in morning Day 2: 10 mg in morning and 10 mg in evening Day 3: 10 mg in morning and 20 mg in evening Day 4: 20 mg in morning and 20 mg in evening Day 5: 20 mg in morning and 30 mg in evening Day 6 and thereafter: 30 mg twice daily
		Psoriatic arthritis	
Roflumilast	a PDE4 inhibitor	Plaque psoriasis	topical use, apply once daily to affected areas
Tapinarof	an AhR agonist	Plaque psoriasis	topical use, apply once daily to affected areas
Spesolimab	an IL-36 receptor antagonist	Pustular psoriasis	a single 900 mg dose by intravenous infusion and an additional intravenous 900 mg dose may be administered one week after the initial dose if flare symptoms persist

### 4.1 TNF-a inhibitors

TNF-a is a crucial factor in immune-related conditions such as RA, AS, inflammatory bowel disease (IBD), psoriasis, and hidradenitis suppurativa (HS). It can be produced by various cells, including DCs, T cells, neutrophils, and keratinocytes. TNF-a inhibitors includes two types: circulating receptor fusion protein (such as etanercept) and monoclonal antibodies (e.g. infliximab, adalimumab, certolizumab pegol and golimumab). For psoriasis, these TNF-a inhibitors can be used alone or with methotrexate to decrease the frequency of antidrug antibodies (ADAs) (96). And they are more suitable to treat psoriatic patients together with IBD or PsA. Certolizumab pegol is effective and well tolerated for women of childbearing potential (97). Reactivation of latent tuberculosis or hepatitis, lymphomas, heart failure and lupus are the severe side effects for TNF-a inhibitors (98).

### 4.2 IL-17 inhibitors

IL-17 is considered as a vital factor during the process of psoriasis because it contributes to the inflammatory response that damages and overturns epidermal keratinocytes (99, 100). Currently, IL-17 inhibitors approved to treat psoriasis include secukinumab, ixekizumab, bimekizumab, and brodalumab. Secukinumab and ixekizumab both act by inhibiting IL-17A, while bimekizumab suppress IL-17A and IL-17F. However, brodalumab selectively binds to the receptor of IL-17. IL-17 inhibitors represent more effective and fast-acting outcomes in patients with psoriasis and have low ADA incidence and low tendency of reactivating tuberculosis. Secukinumab and ixekizumab have also been approved to treat PsA by FDA (101). In a phase 2b trial, significant improvements were achieved by bimekizumab in PsA (102). Since the natural effect of antifungal immunity by IL-17A, long-term use of IL-17 inhibitors may lead to Candida infections (103). Brodalumab was also assumed to be related with suicide, joint pain, and headache (104). Different from secukinumab and ixekizumab only targeting IL-17A, brodalumab inhibits IL-17A, IL-17C, IL-17F, and IL-17E through IL-17RA, representing better efficacy (105).

### 4.3 IL-12/IL-23 p40 and IL-23 p19 inhibitors

The expression of the p40 subunit and p19 subunit in psoriasis is increased (106). The subunit of p40 is commonly shared by IL-12 and IL-23, while p19 is only owned by IL-23. Ustekinumab is a humanized biological antibody neutralizing the subunit p40 in IL-12 and IL-23, treating Crohn’s disease, ulcerative colitis, psoriasis and PsA (107). The subunit of p35 is another component of IL12 and the expression level the p35 subunit was not upregulated in the skin of patients with psoriasis, implying the direct inhibiting of IL-23 is likely to be more effective. To date, biologic agents targeting the p19 subunit of IL-23 involving guselkumab, tildrakizumab, risankizumab, and mirikizumab are approved for psoriasis treatment (108). Guselkumab is shown to be effective in PsA patients without biotherapy experience or previously treated with TNF-α inhibitors (109, 110). Besides, risankizumab has been proven to have much better effects than adalimumab and ustekinumab (28, 111).

### 4.4 Small molecule inhibitors

Small molecule inhibitors refer to chemical substances that can attach themselves to enzymes or proteins, thereby hindering their functions. Small molecule inhibitors have a wide range of applications in medicine, including the treatment of psoriasis.

Janus kinase (JAK) inhibitors are compounds that obstruct the intracellular signaling pathway that is facilitated by JAK and STAT proteins. This interference results in the inhibition of the transcription of proinflammatory cytokines (112). The signaling mechanism of the IL-23 receptor depends on a heterodimer of JAK2 and tyrosine kinase (TYK) 2 for transducing signals, which emphasizes the significance of JAKs in the development of psoriasis and the potential of JAK inhibitors for therapeutic purposes (113). Two JAK inhibitors (tofacitinib and upadacitinib) have been authorized by the FDA to manage PsA (114). Moreover, the FDA granted approval for deucravacitinib in September 2022 for the treatment of moderate-to-severe psoriasis, which is a specific type of JAK inhibitor known as a TYK2 inhibitor.

Phosphodiesterase - 4 (PDE-4) is an enzyme that plays a key role in the regulation of intracellular levels of cyclic adenosine monophosphate (cAMP), resulting in activation of NF-kB and inhibition of CRE-binding protein and ATF-1. PDE-4 inhibitors are a class of drugs that block the activity of PDE-4 enzymes. By inhibiting PDE-4, these drugs increase the levels of cAMP in cells, which has anti-inflammatory effects. Apremilast is an oral PDE4 inhibitor approved for treating patients with moderate to severe plaque psoriasis and PsA (115, 116). A topical agent of PDE-4 inhibitor named roflumilast cream is being investigated for the topical treatment of psoriasis (117).

The Aryl hydrocarbon receptor (AhR) is a transcription factor that is dependent on cytosolic ligands and is expressed in various types of skin cells (118), playing a role in the pathogenesis of inflammatory skin diseases, including psoriasis (119). Tapinarof, an AhR agonist, has recently been approved by the FDA as a non-steroidal topical treatment for plaque psoriasis. Studies have shown that it is effective in treating the condition and has a favorable safety profile (118).

## 5 Conclusions

**Figure 1:**
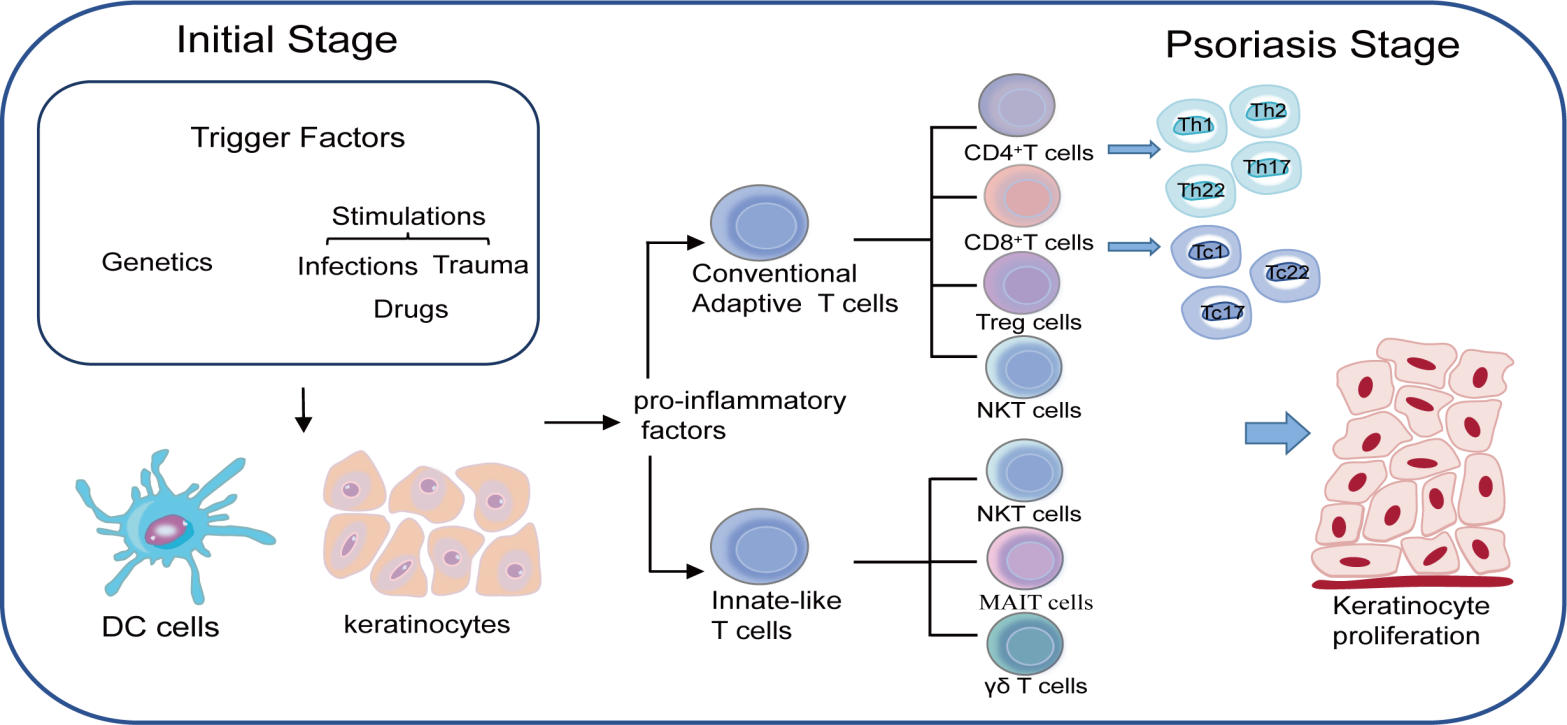
Both genetic susceptibility and environmental triggers such as infections, drugs and trauma play important roles in developing psoriasis. Keratinocytes together with DC cells are responsible for the initial stage through the production of pro-inflammatory factors. T cells are believed to be in the center of accelerating the process of psoriasis as two main subtypes of T cells namely conventional adaptive T cells and innate-like T cells work together to maintain the inflammatory loop during the psoriasis.

T lymphocytes are of utmost importance in the pathogenesis of psoriasis. Great progress on T lymphocytes and their functions in the immune-mediated diseases have been made over the past few years, helping us to understand the pathogenesis of psoriasis more clearly and specifically. Psoriasis is no longer regarded as a Th1 type disease and many other T lymphocytes such as Th1, Th17, Treg and Th22 cells also make contributions to the psoriasis development by interacting with each other (**Figure 1**). Undoubtedly, new types of T lymphocytes may be discovered with the increasingly understandings of the pathogenesis of psoriasis, which will in turn provide novel therapy approaches in the future.

## Author contributions

HW and RW contributed to conception and design of the study. PZ and SL wrote the first draft of the manuscript. HC performed the chart. All authors contributed to the article and approved the submitted version.
